# Characterizing location of spotted lanternfly egg masses in wooded habitat during early invasion stages

**DOI:** 10.3389/finsc.2022.964736

**Published:** 2022-09-02

**Authors:** Katarzyna Madalinska, Robert McDougall, Anne L. Nielsen

**Affiliations:** Rutgers University, Department of Entomology, Bridgeton, NJ, United States

**Keywords:** *Lycorma delicatula*, survey, biosurveillance, invasive species, egg masses, vineyards, early detection

## Abstract

The spotted lanternfly, *Lycorma delicatula* (Hemiptera: Fulgoridae), is an invasive planthopper from Asia that is estimated to have spread 17 km/yr since it's initial detection in Pennsylvania in 2014. *Lycorma delicatula* is a pest to the agricultural and forestry industries in the Mid-Atlantic region of the United States, in part due to its highly polyphagous nature. Current detection relies on visual observations, unbaited traps, or eDNA surveillance in its primary hosts, including grape and hardwoods. These approaches narrow the surveillance area by concentrating on known host plants but could be further refined to narrow the search parameters from the 100+ known host plants. Because *L. delicatula* appears to have a strong population buildup in wooded areas, we evaluated the relationship between egg mass presence and habitat characteristics in wooded habitats adjacent to vineyards in New Jersey at six farms within the first two years of *L. delicatula* detection. Habitat characteristics included distance from wood edge, and presence of a critical host plant *Ailanthus altissima*, and presence of *Vitis* spp. within 4.5 m. We identified a significant relationship between egg mass presence and *Vitis* spp. with an 88% probability of finding an egg mass close to a wild grapevine, dropping to 9% where grapes were absent. During the early invasion stages when this research was conducted, a two-year delay from initial detection in wooded habitats to nymphal presence in the vineyard was observed.

## Introduction

The spotted lanternfly, *Lycorma delicatula* (White) (Hemiptera: Fulgoridae), is an invasive plant hopper native to Northern China (1–3) that was first detected in South Korea in 2004 (4), Japan in 2009 (5), and the United States in 2014 (1). Since its detection in Berks County, Pennsylvania, USA, *L. delicatula* has since spread to several adjacent states including New Jersey in 2018 (6). Recent modeling predicts that *L. delicatula* may become established throughout the Northeast and Pacific Northwest of North America and portions of South America, Europe, Africa, Australia, and Oceania (7).

*L. delicatula* poses a significant threat to the agricultural and forestry industries in the United States (8). They are associated with feeding on 103 host species worldwide in 33 families and 17 orders (9). Nymphs and adults feed gregariously on the phloem from host plants resulting in reduced plant vigor (10), sooty mold (11), wilting, yield loss, and potential plant death (12). In cultivated grapes, adult *L. delicatula* feeding has been associated with cluster reduction (13) and sooty mold (14). *L. delicatula* overwinters in the egg stage within egg masses, ranging from 35 to 50 eggs, which can be oviposited on nearly any organic or inorganic substrate. In Pennsylvania and New Jersey, eggs are laid from September through November in parallel rows and covered with a light brown protective coating (15, 16).

Invasive species can be detrimental to native ecosystems, reduce biodiversity, and negatively impact the economy and human health (17–20). Early detection has been an effective tool in controlling and potentially eradicating invasive species (20). However, once invasive populations have been established, complete eradication may no longer be achievable and management tactics such as quarantine regulations, barrier zones, and surveillance (21) should be implemented to impede the spread. Previously successful examples of invasive species management include the light brown apple moth, *Epiphyas postvittana* (Walker) (Lepidoptera: Tortricidae) in New Zealand (22) and the spongy moth, *Lymantria dispar* (Linnaeus) (Lepidoptera: Erebidae) in the United States (23). Egg masses were found to be a viable surveillance option for nascent *L. dispar* populations and to delimit surveys for management decisions (23, 24). Egg mass deposition of *L. delicatula* resembles that of *L. dispar* in both appearance and placement. A similar surveillance program for *L. delicatula* may be appropriate as it has spread beyond established quarantine zones with populations large enough to warrant management.

Monitoring and surveillance of *L. delicatula* currently relies on detection during their active life stages primarily with the use of sticky bands or modified circle trunk traps (25, 26). These methods rely on intercepting individuals climbing up a host plant as there is no species-specific lure for *L. delicatula*. Baiting sticky bands with host plant volatiles (E,E)-α-farnesene, methyl salicylate, and (Z)-3-hexenol volatiles had mixed results in the field and may not increase detection of *L. delicatula* (27). Environmental DNA (eDNA) surveillance (28) has successfully detected invasive insects, including *L. delicatula*, by picking up trace amounts of DNA left behind in terrestrial environments and does not require visual inspection for insects (29–31). While effective during the spring, summer, and fall, these surveillance methods are not yet able to detect *L. delicatula* during the dormant egg stage. As egg masses can lead to population hot spots early during the invasion process, we evaluated habitat characteristics in wooded sites associated with *L. delicatula* egg masses.

Forests in the Mid-Atlantic region have become more fragmented due to increases in agricultural, residential, and urban development over recent decades (32). Fragmentation of the landscape has resulted in reduced patch size and increased forest edge (32) and fragments frequently border vineyards in New Jersey. These wooded areas are habitat for multiple life stages of *L. delicatula* throughout their univoltine lifespan (33). Oviposition begins in late September and continues through November. This study evaluates the habitat characteristics associated with *L. delicatula* egg masses within wooded habitats during the early invasion stages. Based on the previous *L. delicatula* phenology research, variables included distance from wood edges (6) and presence of key host plants (wild grape - *Vitis* spp. and the invasive Tree of Heaven (ToH)- *Ailanthus altissima*) (14). We aim to predict *L. delicatula* future pressure within vineyards by measuring nymphal presence and abundance in the following year.

## Methods

### Sites

**Figure 1:**
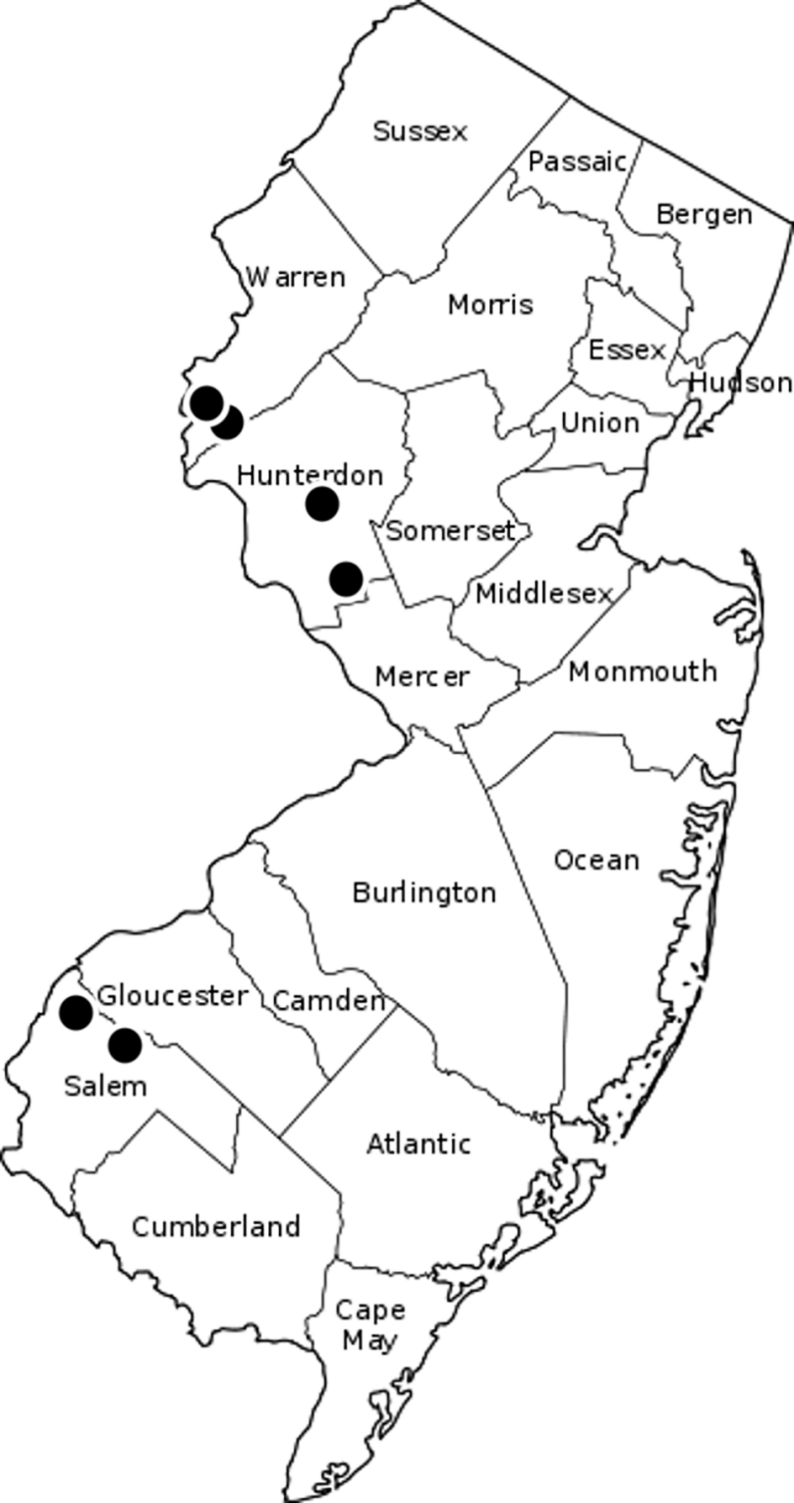
Map of New Jersey with the six sample sites.

Surveys were conducted at commercial vineyards with at least a partial wooded habitat in Pedricktown, Pilesgrove, Pittstown, Ringoes, and two farms in Milford (A and B), New Jersey. *L. delicatula* was observed in 2018 within the vineyards Milford A and Pittstown and in 2019 in all other locations (**Figure 1**).

### Wooded area survey

Each site was surveyed once between January and March of 2020 when egg mass visibility is greatest due to decreased foliage. At each site, the primary hardwood tree species were recorded, the size of the sample area was determined through GoogleEarth, and transects spaced 15.2 m apart were established, with the number of transects varying based on the nature and extent of the wooded area at each site (**Table 1**). This resulted in four transects at Milford A and Pilesgrove, five transects at Milford B, Pedricktown, and Pittstown, and six transects at Ringoes, NJ. Within each transect, sample trees were haphazardly selected at 15.2 m intervals beginning at the wood edge and extending 30.5 – 137.2 m into the woods for a total of 157 sample points. The number of egg masses, texture of tree (trees with peeling or smooth bark), and diameter at breast height (DBH) was recorded for each sample. An additional three-minute visual survey recorded *L. delicatula* egg masses on the surrounding vegetation within a 4.5 m radius of the sample tree. Observations were limited to a height of 3.0 m to ensure accurate identification of egg masses. Observations at 3.0 m represent 1.78% of the density of egg masses expected to be present on the sampled tree (34). Presence or absence of *Vitis* spp. and *A. altissima* within the 4.5 m radius were recorded.

**Table 1:** Wooded habitat plot characteristics of New Jersey sampling sites.

Sample Site	Coordinates	Detection Year	Plot Size	# of Transects	# of Samples	Proportion of Samples with *Vitis*	Proportion of Samples with *Ailanthus*	Dominant Hardwood Species	Mean # of *L. delicatula* Egg Masses/Tree	SEM	Distance from Vineyard Edge
Milford A	40°36’55” N 75°09’49” W	2018	4645 m^2^	5	25	0.72	0.16	black walnut (*Juglans nigra*), sycamore (*Platanus occidentalis*), eastern red cedar (*Juniperus virginiana*)	6.68	(± 1.77)	62.5 m
Milford B	40°35’12” N 75°09’34” W	2019	2787 m^2^	4	16	0.69	0	black walnut (*Juglans nigra*), eastern red cedar (*Juniperus virginiana*)	1.69	(± 0.51)	5.5 m
Pedricktown	39°45’42” N 75°24’42” W	2019	6271 m^2^	5	29	0.24	0	walnut (*Juglans* spp.), black cherry (*Prunus serotina*), sweet gum (*Liquidambar styraciflua*), maple (*Acer* spp.)	2.14	(± 1.20)	7.9 m
Pilesgrove	39°41’16” N 75°22’05” W	2019	2323 m^2^	4	14	0.36	0.43	tree of heaven (*Ailanthus altissima*), Callery pear (*Pyrus calleryana*)	1.07	(± 0.52)	60.4 m
Pittstown	40°34’15” N 74°58’34” W	2018	10452 m^2^	5	50	0.32	0	oak (*Quercus* spp.), witch hazel (*Hamamelidaceae* spp.), elm (*Ulmus* spp.)	1.1	(± 0.53)	122.2 m
Ringos	40°25’34” N 74°49’41” W	2019	4181 m^2^	6	23	0.48	0	black walnut (*Juglans nigra*), hickory (*Carya* spp.), elm (Ulmus spp.)	1.35	(± 0.50)	7.3 m

### Vineyard survey

Surveys for *L. delicatula* early life stages within the vineyard area closest to the woods were conducted at Milford A, Pilesgrove, and Pittstown, NJ on 26 May, 11 June, 25 June, and 9 August 2020. Egg mass counts were limited to the vines themselves since no additional host plants were present within the vineyard. Five survey transects occurred within the vineyard with sample points (i.e., grapevine) starting at the perimeter (0 m) and continuing at 15.2 m intervals up to 76.2 m into the vineyard. The number of egg masses within each ~6 vine sample were recorded prior to hatch. Following hatch biweekly sampling was conducted when each of the four nymphal stages were most prominent within the vineyard for a total of four time points (time point 1= primarily 1^st^ instars, time point 2 = primarily 2^nd^ instars, time point 3 = primarily 3^rd^ instars, and time point 4 = primarily 4^th^ instars).

### Data analysis

Egg mass presence/absence and density in the wooded area by site were analyzed, with distance from edge, the presence/absence of *Vitis* spp., presence/absence of *A. altissima*, tree bark texture, and DBH used as explanatory variables. Data were analyzed with a generalized linear mixed model (GLMM) with egg presence fitted to a logistic model with binomial distribution and egg mass density fitted to a generalized linear model with Poisson distribution. GLMMs were produced using the R package glmmTMB (35). Each response variable was tested against all five possible explanatory variables, alone and in all possible combinations, for a total of 27 models each, each also containing study site (vineyard) as a random variable. A null model was also produced (with a constant in place of any fixed explanatory variables). Models were ranked based on their akaike information criterion (AIC) values, with models with lower AIC values ranked more highly (36). The highest ranked model, and all models with an AIC value within ΔAIC <2 of that model, were compared to the null model using an ANOVA with chi-squared test to determine if they were significantly different to the null model. R^2^ values of these models were determined using the r2_nakagawa function from the R Package DHARMA Data analysis was performed using R version 4.0.2 (37).

The average number of egg masses during 3-minute visuals per sample at each site were analyzed with a general linear model with Poisson distribution and log-link function (38) to categorize invasion history into “new” (*L. delicatula* in high densities) or “established” (*L. delicatula* in low densities) groups. In the vineyard survey analysis of *L. delicatula* nymphs was restricted to the first two sample points because they more accurately represent the relationship with egg mass deposition and its proximity to the wooded edge. Data did not meet assumptions of normality and was log(x+1) transformed. Transformed data was analyzed with a two-way ANOVA for distance and invasion history and the interaction. Data analysis was performed using JMP Pro 15 (2019) (38).

## Results

### Wooded area survey

A total of 357 egg masses were counted within the wooded habitats across all six sites within the first two years of *L. delicatula* detection in New Jersey. The average number of egg masses was 2.52 ( ± 0.41) per tree (**Table 1**). Two of the sites had *L. delicatula* sightings in 2018 and the remaining four reported sightings in 2019.

The most highly ranked model examining presence or absence of egg masses in wooded habitat was one that showed egg masses to be significantly more likely to be found in presence of *Vitis* spp., and in the presence of trees with peeling bark (p<0.001). All sites had *Vitis* spp. in the survey area, but not all sites had *A. altissima*. The 13 most highly ranked models were all 13 models that contained *Vitis* presence as an explanatory variable (**Table 2**).

**Table 2:** Presence of *Lycorma delicatula* egg masses in New Jersey wooded habitats as influenced by environmental variables.

Model Number	Environmental Variables	AIC	P-value	R^2^ (marginal)	Direction
14	Bark, Grape	84.4	<0.001	0.586	positive, positive
25	Grape	85.4	<0.001	0.561	positive, positive
20	Grape, Distance	86	<0.001	0.578	positive
7	Bark, ToH, Grape	86.3	<0.001	0.597	positive, negative, positive
17	ToH, Grape	86.8			
10	ToH, Grape, Distance	87.3			
21	Grape, DBH	87.3			
2	Bark, ToH, Grape, Distance	87.5			
12	Grape, Distance, DBH	87.7			
5	Bark, Grape, Distance, DBH	87.8			
3	Bark, ToH, Grape, DBH	88.2			
11	ToH, Grape, DBH	88.7			
6	ToH, Grape, Distance, DBH	89.1			
1	ToH, Grape, Distance, DBH, Bark	89.3			
16	Bark, DBH	166.5			
23	Bark	166.8			
9	Bark, ToH, DBH	167.1			
13	Bark, ToH	167.2			
Null		167.7			
15	Bark, Distance	168			
27	DBH	168			
8	Bark, ToH, Distance	168.3			
24	ToH	168.3			
26	Distance	168.4			
4	Bark, ToH, Distance, DBH	168.6			
19	ToH, DBH	168.9			
18	ToH, Distance	169			
22	Distance, DBH	169.1			

**Figure 2:**
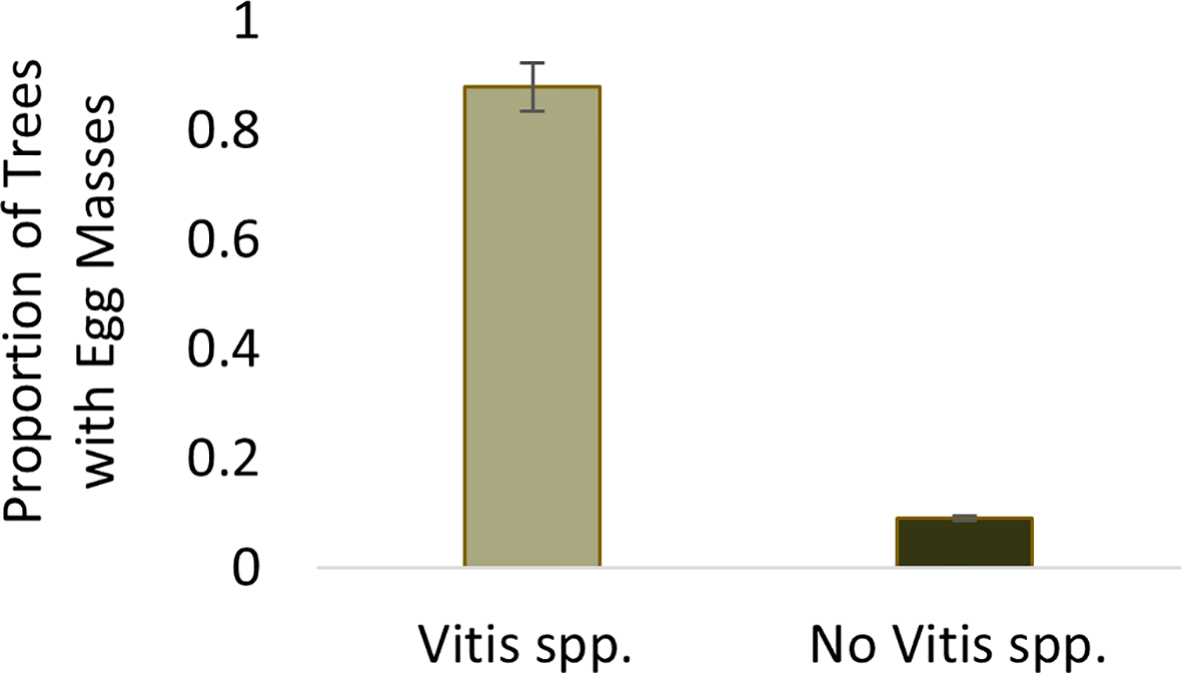
Proportion of sample trees containing *Lycorma delicatula* egg masses within wooded areas near vineyards in New Jersey with (0.88) and without (0.09) the presence of *V.* spp.

The probability of finding at least one egg mass at a sample point where *Vitis* spp. was present was 0.88. The probability of finding an egg mass decreased significantly (Wilcoxon test, W=3351.5, p<0.001) to 0.09 at sample trees where *Vitis* spp. was not present (**Figure 2**).

The relationship between egg mass density and the measured environmental variables was less clear. The most highly ranked model was one that showed a positive relationship between egg mass number and *Vitis* presence, wood edge distance, and sample tree DBH. No other models were within ΔAIC <2 of this model, however 26 of the 27 models tested ranked more highly than the null model (**Table 3**)

**Table 3:** The effect of environmental variables on density of *Lycorma delicatula* egg mass in New Jersey.

Model Number	Variables	AIC	P	R2 (marginal)	Direction
12	Grape, Distance, DBH	421.8	<0.001	0.533	positive, positive, positive
6	ToH, Grape, Distance, DBH	423.8			
1	ToH, Grape, Distance, DBH, Bark	423.9			
2	Bark, ToH, Grape, Distance	451.6			
20	Grape, Distance	451.9			
10	ToH, Grape, Distance	453.1			
4	Bark, ToH, Distance, DBH	469.3			
22	Distance, DBH	470.1			
11	ToH, Grape, DBH	478.1			
3	Bark, ToH, Grape, DBH	479			
21	Grape, DBH	485			
18	ToH, Distance	495.2			
8	Bark,ToH, Distance	495.4			
26	Distance	495.4			
15	Bark, Distance	496.5			
7	Bark, ToH, Grape	501.4			
17	ToH, Grape	502.2			
19	ToH, DBH	504.3			
25	Grape	505.1			
9	Bark, ToH, DBH	505.5			
14	Bark, Grape	506.1			
5	Bark, Grape, Distance, DBH	506.4			
16	BarkDBH	516.1			
27	DBH	517.1			
24	ToH	526.5			
13	Bark, ToH	526.8			
Null		535.5			

### Occurrence within vineyard

Using density of egg masses from each site within wooded areas as a parameter, adjacent vineyards were divided into two categories: 1) “established” *L. delicatula* populations with egg mass densities above three per sample site (Milford A) and 2) “new” belonging to all other farms with egg mass densities below 3 per sample point (*x* ^2 =^ 21.58, df=5, *P*=0.006). An average of 1.11 ( ± 0.27) egg masses were found within the wooded area of the vineyards with a “new” *L. delicatula* invasion history and an average of 6.68 ( ± 1.77) for the “established” population. Using this categorization, there was a significant model effect (F=3.30, df=9, 74, *P=*0.002) for nymph occurrence the following year in vineyards. In the Milford A “established” site we recorded a positive correlation between time point 1 and nymphs with egg masses (y= 18.916x – 20.759, R² = 0.705). A negative correlation was found in time point 2 with egg masses as 2^nd^ instars dispersed (y=-5.812x + 200.68, R² = 0.043). No correlation was found in time point 3 (y = 1.033x + 36.363, R² = 0.023) and time point 4 (y = -0.067x + 0.848, R² = 0.321).

**Figure 3:**
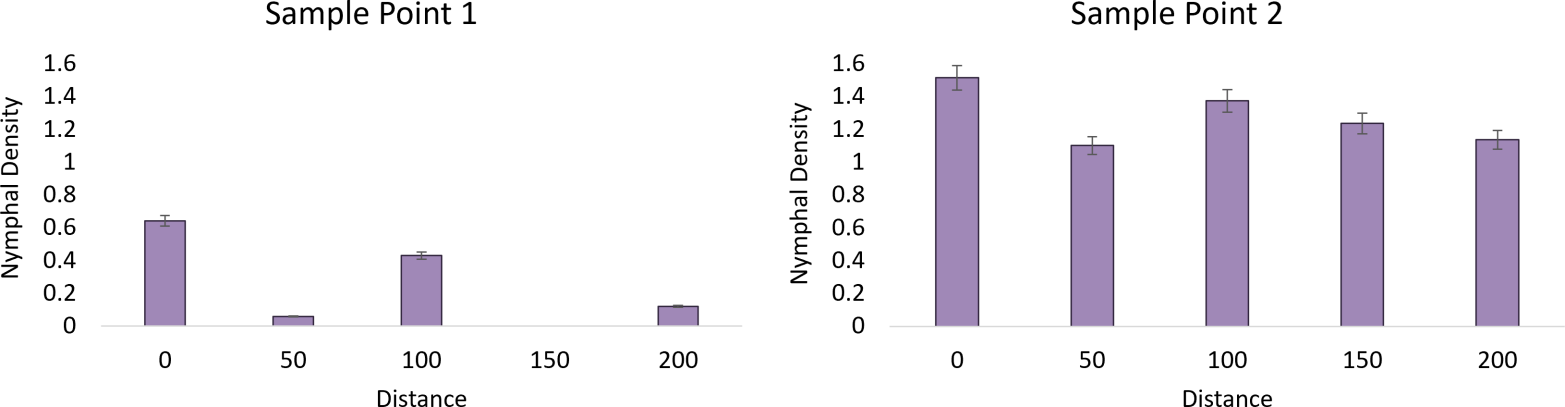
The average nymphal density of *L. delicatula* within New Jersey vineyard at sampling point 1 on 26 May 2020 (1^st^ instars) and sampling point 2 on 11 June 2020 (2^nd^ instars) in relation to distance looking at vineyards with “established” populations. No nymphs were found at “new” sites.

Analysis of the first time point at the “established” vineyard suggests that nymph density (F=15.97, df=1, *P*=0.0002), distance (F=2.92, df=4, *P*=0.028), and their interaction were significant (F=2.92, df=4, *P*=0.0276) and demonstrated a strong edge effect (**Figure 2**). The second sampling point showed a significant effect of nymph density (F=127, 73, df=1, *P*<0.0001) but not distance (F=1.64, df=4, P=0.175), suggesting that the edge effect had diminished as the nymphs dispersed away from their egg mass. No nymphs were observed within the “new” sites in 2020 for analysis (**Figure 3**).

## Discussion

This study evaluated habitat characteristics consistent with the presence and density of *L. delicatula* egg masses in wooded habitats and their relationship to presence of nymphs within vineyards the following year. *L. delicatula* are in their egg stage for six months of the year and, therefore, this is an important life stage for detection of nascent populations. We found that the number of egg masses within sample points in wooded habitats were positively correlated with the presence of wild *Vitis* spp., which may help delimit biosurveillance efforts and management during the spread phase.

Egg mass surveys have been used to effectively manage *L. dispar* spread using egg mass densities to identify satellite populations prior to hatch (21, 23). The indiscriminate nature of *L. delicatula* egg mass oviposition makes identifying habitat features and variables associated with egg masses critical to effective surveillance. We found that distance from edge had a small effect on egg mass density but the presence of *Vitis* spp. most strongly predicted the presence of egg masses 88% of the time despite not being a preferential substrate for oviposition itself. Surveyed sites varied with available plant species for oviposition. *Vitis* spp. was the only host consistently present at all sampling sites. However, *Vitis* spp. distribution within these wooded habitats was not uniform. Identifying the association of egg mass presence with *Vitis* spp. can help isolate surveillance efforts to parts of the wooded habitat where *Vitis* spp. is most concentrated. Although *A. altissima* has been identified as a preferential oviposition site (39), the presence of this host plant at our sites was inconsistent and its effects less impactful than those of *Vitis* spp. As Liu and Hartlieb (40) found, no cardinal preference was observed, rather egg mass placement varied depending on host characteristics. Sample tree DBH ranged from 2.5 cm to 343 cm across the six sites. Trees with narrow trunk diameter had egg masses oviposited near the base, while larger diameter trees exhibited egg masses primarily on the underside of branches. Trees with peeling or flaking bark displayed indiscriminate oviposition of egg masses within bark crevices throughout the trunk.

We found that within the early stages of *L. delicatula* invasion, egg mass densities within wooded habitats have an impact on *L. delicatula* nymphal presence within vineyards the following year. Five of the six sites had an average of 1.11 ( ± 0.27) egg masses per tree while Milford A had 6.68 ( ± 1.77) egg masses per tree. At Milford A (“established”), we observed a spillover from the wooded habitat as seen in egg mass and nymphal populations in the vineyards the following Spring. This suggests that there is about a two-year lag from first detection of egg masses in wooded habitats and presence of nymphs in the vineyard.

## Conclusion

Early detection tools are key to decreasing the potential spread of invasive species. The presence of trees with peeling bark was positively associated with egg masses, which suggests that biosurveillance efforts at early invasion stages of *L. delicatula* should include the entire wooded habitat but can be focused on trees with peeling bark near wild grape. While *Vitis* spp. is a known host plant of *L. delicatula*, and an at-risk agricultural crop, this is the first study to highlight the association of *Vitis* spp. with egg mass oviposition. There was a two-year lag before nymphs were observed in the adjacent commercial vineyards, which provides time for mitigation efforts to delay or decrease the impact to cultivated grapes.

## Data availability statement

The raw data supporting the conclusions of this article will be made available by the authors, without undue reservation.

## Author contributions

KM and AN designed experiments, collected and analyzed the data, and wrote the paper. RM contributed to the data analysis. All authors contributed to the article and approved the submitted version.

## Funding

This research was funded through the USDA SCRI 2019 2019-51181-30014 and USDA APHIS FarmBill Suggestion PPA 7721.

## Acknowledgments

Thank you to the grower collaborators who participated in this survey as well as Ann Rucker and Kara Ladle for assisting in the field data collection for this survey.

## Conflict of interest

The authors declare that the research was conducted in the absence of any commercial or financial relationships that could be construed as a potential conflict of interest.

## Publisher’s note

All claims expressed in this article are solely those of the authors and do not necessarily represent those of their affiliated organizations, or those of the publisher, the editors and the reviewers. Any product that may be evaluated in this article, or claim that may be made by its manufacturer, is not guaranteed or endorsed by the publisher.
